# Inhibition of autophagy blocks cathepsins–tBid–mitochondrial apoptotic signaling pathway via stabilization of lysosomal membrane in ischemic astrocytes

**DOI:** 10.1038/cddis.2017.34

**Published:** 2017-02-16

**Authors:** Xian-Yong Zhou, Yu Luo, Yong-Ming Zhu, Zhi-He Liu, Thomas A Kent, Jia-Guo Rong, Wei Li, Shi-Gang Qiao, Min Li, Yong Ni, Kazumi Ishidoh, Hui-Ling Zhang

**Affiliations:** 1 Jiangsu Key Laboratory of Translational Research and Therapy for Neuro-Psycho-Diseases, College of Pharmaceutical Science; Department of Pharmacology and Laboratory of Cerebrovascular Pharmacology; Jiangsu Key Laboratory of Preventive and Translational Medicine for Geriatric Diseases, School of Public Health, Soochow University, Suzhou, China; 2 Guangzhou Institute of Traumatic Surgery, Guangzhou Red Cross Hospital, Medical College, Jinan University, Guangzhou, China; 3 Stroke Outcomes Laboratory, Department of Neurology, Baylor College of Medicine, Houston, TX, USA; 4 Center for Translational Research on Inflammatory Diseases, Michael E DeBakey Veterans Affairs Medical Center, Houston, TX, USA; 5 Institute for Health Sciences, Division of Molecular Biology, Tokushima Bumi University, Yamashiro-cho, Tokushima City, Tokushima, Japan

## Abstract

Our previous study and others have demonstrated that autophagy is activated in ischemic astrocytes and contributes to astrocytic cell death. However, the mechanisms of ischemia-induced autophagy remain largely unknown. In this study, we established a rat's model of permanent middle cerebral artery occlusion (pMCAO) and an *in vitro* oxygen and glucose deprivation (OGD) model. Autophagy was inhibited by either pharmacological treatment with 3-methyladenine (3-MA) and wortmannin (Wort) or genetic treatment with knockdown of *Atg*5 in primary cultured astrocytes and knockout of *Atg*5 in mouse embryonic fibroblast (MEF) cells, respectively. We found that pharmacological or genetic inhibition of autophagy reversed pMCAO or OGD-induced increase in LC3-II, active cathepsin B and L, tBid, active caspase-3 and cytoplastic cytochrome c (Cyt-c), and suppressed the injury-induced reduction in mitochondrial Cyt-c in ischemic cortex, in injured astrocytes and MEF cells. Immunofluorescence analysis showed that 3-MA or Wort treatment reversed OGD-induced release of cathepsin B and L from the lysosome to the cytoplasm and activation of caspase-3 in the astrocytes. Furthermore, treatment of 3-MA or Wort decreased OGD-induced increase in lysosomal membrane permeability and enhanced OGD-induced upregulation of lysosomal heat shock protein 70.1B (Hsp70.1B) in astrocytes. Inhibition of autophagy by 3-MA or Wort reduced infarction volume in rats and protected OGD-induced astrocytic cell injury. A non-selective caspase inhibitor z-VAD-fmk or a specific caspase-3 inhibitor Q-DEVD-OPh also rescued OGD-induced astrocytic cell injury. In conclusion, our presenting data suggest that inhibition of autophagy blocks cathepsins–tBid–mitochondrial apoptotic signaling pathway via stabilization of lysosomal membranes, possibly due to upregulation of the lysosomal Hsp70.1B in ischemic astrocytes.

Historically, three main morphological types of programmed cell death have been identified: type I apoptotic cell death, type II autophagic cell death and type III, which includes necrosis and cytoplasmic cell death.^1^ Currently, there is no approved neuroprotective agent for acute ischemic stroke. One of the reasons may be due to the multiplicity of cell death mechanisms in which inhibition of a particular mechanism leaves the brain vulnerable to the alternative ones. ^2^ Therefore, it is essential to understand the different cell death mechanisms and their interactions.^2^ Autophagy is a highly regulated process involving the bulk degradation of cytoplasmic macromolecules and organelles in mammalian cells via the lysosomal system, and is essential to the long-term health of cells, including neurons.^3^ Autophagy contributes to both cell survival and cell death.^3^ In recent years, the importance of autophagy in some human diseases has received much attention.^4, 5, 6^

In the context of cerebral ischemia, it is proposed that autophagy is protective.^7, 8^ But increasing evidence indicates that autophagy is activated and involved in neuronal death in different animal models of ischemic brain injury, including hypoxia,^9^ global^10^ and focal ischemia.^11^ Accumulating reports have shown that autophagy and apoptosis appear to interact with each other either positively or negatively under certain conditions.^12, 13, 14^ Lysosomal proteases associated with autophagy, such as cathepsin B, have a role in apoptosis via cleavage of Bid, release of cytochrome c (Cyt-c) and activation of caspases in both neurons and non-neural cells.^15, 16^ Therefore, cathepsins may have important roles in the crosstalk between apoptosis and autophagy.^12^

Stroke leads to the death or injury of both neurons and astrocytes. Astrocytes are involved in a number of activities that profoundly influence the consequences of ischemic brain damage, including the maintenance of water balance and the blood–brain barrier, the regulation of cerebral blood flow, brain energetics, glutamate and ion homeostasis, inflammation, trophic factor production, neurogenesis and vasculogenesis.^17, 18, 19, 20, 21, 22, 23^ Therefore, the protection of astrocytes is likely required for neuronal survival and for functional recovery after ischemic stroke.^23, 24^ Astrocyte has been considered as a promising target for novel therapeutic approaches in ischemic brain protection.^12, 24^ Astrocyte apoptosis and autophagy may contribute to the pathogenesis of cerebral ischemia.^12, 24, 25, 26, 27, 28^ The crosstalk between apoptosis and autophagy is largely unknown in ischemic astrocyte injury.

In 2010, we provided the first evidence that autophagy is activated in ischemic astrocytes and contributes to astrocytic cell death.^12^ More recently, we further found that the activation of the cathepsin B and L and their movement into the cytosol contribute to ischemia-induced astrocytic cell injury via tBid–mitochondrial apoptotic signaling pathways.^24^ It is established that the movement of cathepsin B or L into the cytosol can be used as a measure of lysosomal membrane permeability (LMP) in neurons or in astrocytes.^24, 29^ In this study, we tested the hypothesis that autophagy activation may induce cathepsins–tBid–mitochondrial apoptotic signaling pathway via increasing the LMP and that this activation may involve the lysosomal Hsp70.1B-dependent regulating mechanism.

## Results

### Inhibition of autophagy reduces infarct volume and blocks ischemia-induced activation of cathepsin B and cathepsin L–tBid–mitochondrial apoptotic signaling pathway in ischemic cortex

Our previous data and others showed that 3-methyladenine (3-MA) treatment at 300–600 nmol (intracerebroventricular (icv)) could reduce infarct volume and improve neurological deficits in rat models of permanent middle cerebral artery occlusion (pMCAO).^11, 12^ In the current study, we confirmed that 3-MA treatment at 300–600 nmol (icv) could decrease infarct volume at 24 h after pMCAO in rats (Supplementary Figure S1). Our previous study further demonstrated that 3-MA treatment at 300–600 nmol (icv) could protect astrocytes injury in the ischemic cortex.^12^

Owing to the ability to induce Bak and Bax, Bid has a key role in apoptotic signaling pathway and results in Cyt-c release.^30^ Our previous study demonstrated that cathepsin B and L were activated in the ischemic cortex after pMCAO, and led to the activation of tBid–mitochondrial apoptotic signaling pathway.^24^ The peak for cathepsin B and L activation was at 6 and 3 h post-ischemia, respectively. The activation of cathepsin B and L led to a maximal increase in tBid, cytoplastic Cyt-c and active caspase-3, and conversely, a maximal reduction in mitochondrial Cyt-c during 12–24** **h post-ischemia period.

**Figure 1:**
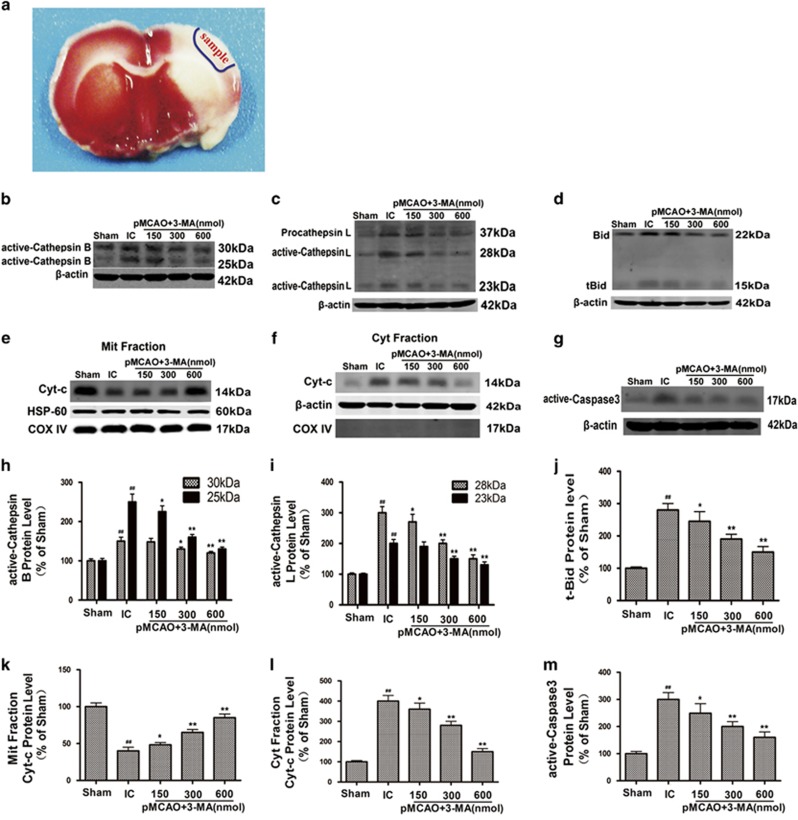
3-MA treatment inhibits ischemia-induced activation of cathepsin B or cathepsin L–tBid–mitochondrial apoptotic signaling pathway in the ischemic cortex. 3-MA (150, 300 and 600 nmol) or vehicle was administrated icv 10 min after ischemia induced by pMCAO. (**a**) Brain slice stained with 2, 3, 5- triphenyltetrazolium chloride (TTC). The region within the blue line indicates the part of the ischemic brain cortex collected as samples for western blotting analysis. (**b-g**) Representative western blotting images of the protein levels of active cathepsin B (**b**) at 6 h or cathepsin L (**c**) at 3 h, tBid (**d**), mitochondrial (**e**) and cytoplastic Cyt-c (**f**) and active caspase-3 (**g**) at 24 h after ischemia. (**h-m**) Columns represent quantitative analysis of immunoblots in (**b**-**g**), respectively (means±S.D., *n*=3). Cyt-c oxidase IV (COX IV), which is located in the inner mitochondrial membrane acts as a mitochondrial marker. *β*-Actin or HSP-60 was used as a loading control. ^##^*P*<0.01 *versus* Sham group; *P<0.05, **P<0.01 *versus* ischemic control group (IC)

In this study, we found that 3-MA treatment at 300–600 nmol (icv) inhibited ischemia-induced increase in active cathepsin B (Figures 1b and h) and cathepsin L (Figures 1c and i) at 6 and 3 h post-ischemia, respectively; and ischemia-induced increase in tBid (Figures 1d and j), cytoplastic Cyt-c (Figures 1f and l) and active caspase-3 (Figures 1g and m), and reduction in mitochondrial Cyt-c (Figures 1e and k) at 24 h after ischemia. These data suggest that ischemia-induced autophagy activation contributes to activation of cathepsin B and L, cleavage of Bid, translocation of Cyt-c from the mitochondria to the cytosol and activation of caspase-3 in the ischemic cortex.

### Inhibition of autophagy protects astrocytes and mouse embryo fibroblast cells against OGD-induced injury

**Figure 2:**
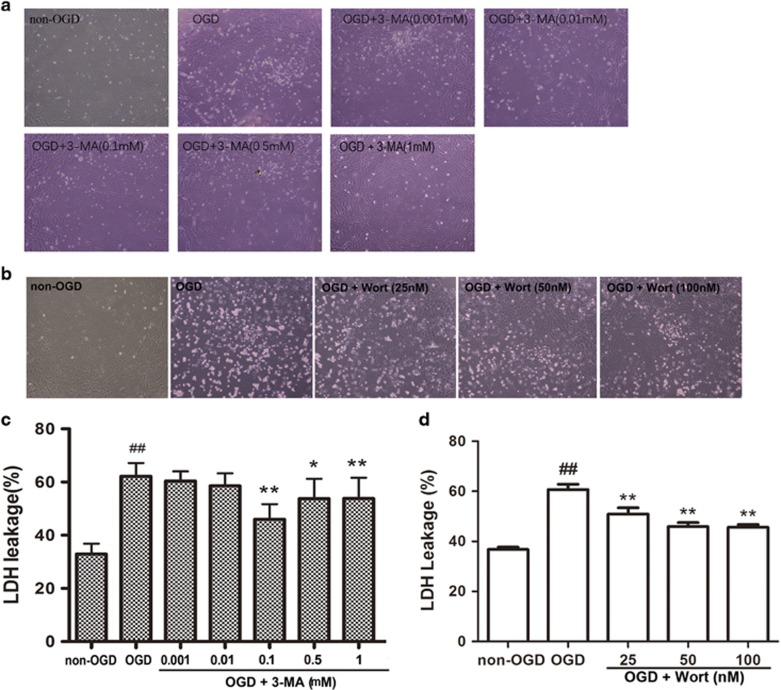
Inhibition of autophagy protects astrocytes against OGD injury. Astrocytes were treated with 3-MA (0.1, 0.5 or 1 mM) or Wort (25, 50 or 100 nM), then underwent OGD treatment for 12 h. (**a** and **b**) Representative light microscope images of astrocytes without OGD or with OGD treatments. (**c** and **d**) LDH leakage analysis showed that 3-MA (**c**) or Wort (**d**) treatment decreased the LDH leakage of astrocytes with OGD treatment. Means±S.D., *n*=6. ^##^*P*<0.01 *versus* non-OGD group; *P<0.05, **P<0.01 *versus* OGD group

We next determined whether inhibition of autophagy could protect astrocytes against oxygen and glucose deprivation (OGD)-mediated injury. Our previous study and others found that a higher dose of 3-MA (10 mM) could inhibit OGD, TNF and palmitic acid-induced autophagy in astrocytes,^12^ in FADD-deficient Jurkat cells^31^ and in endothelial cells. This dose also exhibited a mild protection against OGD-induced injury to astrocytes and a significant protection against palmitic acid-induced injury to endothelial cells. In this study, we further found that lower doses of 3-MA (0.1, 0.5 or 1 mM) also could protect astrocytes against OGD-induced injury. Figure 2 showed that the number of astrocytes appeared to increase (Figure 2a) and the leakage of LDH was decreased (Figure 2c) in the OGD+3-MA group, compared with these in the OGD group. Moreover, wortmannin (Wort), another autophagy inhibitor, also appeared to increase the number of astrocytes (Figure 2b) and to decrease leakage of LDH (Figure 2d). 3-MA (0.1, 0.5 or 1 mM) or Wort (25, 50 or 100 nM) alone did not alter the number of astrocytes (data not shown) and the leakage of LDH (data not shown). These results indicate that 3-MA or Wort at these concentrations are not toxic in cultured astrocytes. The above results show that the inhibition of autophagy protects astrocytes against OGD-induced injury.

In support of the above results, we found that inhibition of autophagy also protected mouse embryo fibroblast cells against OGD injury. Compared with these in the OGD+Atg5+/+ group, the number of mouse embryo fibroblast cells was significantly increased and the leakage of LDH was markedly decreased in the OGD+Atg5−/− group (Supplementary Figures S2a and b).

### Inhibition of autophagy blocks OGD-induced activation of cathepsin B and cathepsin L–tBid–mitochondrial apoptotic signaling pathway in astrocytes and mouse embryo fibroblast cells

**Figure 3:**
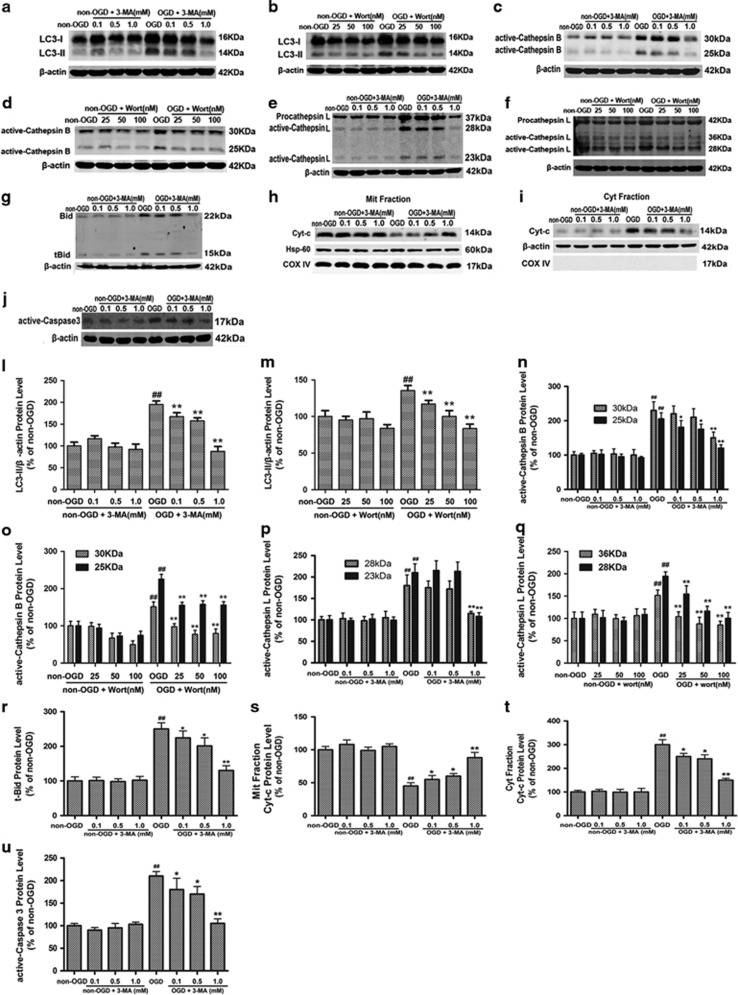
Inhibition of autophagy blocks OGD-induced activation of cathepsin B or cathepsin L–tBid–mitochondrial apoptotic signaling pathway in astrocytes. 3-MA (0.1, 0.5 or 1 mM), Wort (25, 50 or 100 nM) or z-VAD (25, 50 or 100 *μ*M) was added in cells 30 min, 2 h or 1 h before OGD, respectively. (**a-j**) Representative western blotting images of the protein levels of LC3-II (**a** and **b**), active cathepsin B (**c** and **d**) at 6 h or cathepsin L (**e** and **f**) at 3 h, tBid (**g**), mitochondrial (**h**) and cytoplastic Cyt-c (**i**) and active caspase-3 (**j**) at 12 h after OGD. (**l-u**) Columns represent quantitative analysis of immunoblots in (**a-j**), respectively (means±S.D., *n*=3). COX IV acts as a mitochondrial marker. *β*-Actin or HSP-60 was used as a loading control. ^##^*P*<0.01 *versus* non-OGD group; *P<0.05, **P<0.01 *versus* OGD group

We next tested the effects of pharmacological or genetic inhibition of autophagy on OGD-induced activation of cathepsin B and cathepsin L–tBid–mitochondrial apoptotic signaling pathway. First, we confirmed that 3-MA (0.1, 0.5 or 1 mM) or Wort (25, 50 or 100 nM) treatment significantly decreased OGD-induced increase in the LC3-II levels in astrocytes (Figures 3a and l; Figures 3b and m). Application of shRNA Atg5 in astrocytes or use of Atg5−/− in mouse embryo fibroblast cells decreased or depleted the expression of ATG5 and LC3-II levels (Supplementary Figures S3a and g, b and h, d and j, e and k; Supplementary Figures S4a and i, b and j). These data indicate that 3-MA at 0.1–1.0 mM, Wort at 25–100 nM, shRNA Atg5 or Atg5−/− treatment inhibits autophagy in astrocytes and in mouse embryo fibroblast cells.

Our recent study demonstrated that cathepsin B and L were activated after OGD-induced astrocyte injury, resulting in the activation of tBid–mitochondrial apoptotic signaling pathway.^24^ The peak for cathepsin B or L activation was at 6 or 3 h post-OGD, respectively; and the maximal increase in tBid, cytoplastic Cyt-c, active caspase-3 and the maximal reduction in mitochondrial Cyt-c were at 12 h post-OGD. In this study, we found that 3-MA (0.1, 0.5 or 1 mM), Wort (25, 50 or 100 nM) or shRNA Atg5 treatment inhibited OGD-induced increase of active cathepsin B (Figures 3c and n, d and o, Supplementary Figures S3c and i) and cathepsin L (Figures 3e and p, f and q; Supplementary Figures S3f and l) at 6 or 3 h post-OGD. These treatments also increased tBid (Figures 3g and r), cytoplastic Cyt-c (Figures 3i and t) and active caspase-3 (Figures 3j and u) and reduced mitochondrial Cyt-c (Figures 3h and s) at 12 h post-OGD. These results indicate that OGD-induced autophagy activates cathepsin B and L, cleaves Bid, releases Cyt-c from the mitochondria to the cytoplasm and activates caspase-3 in ischemic astrocytes.

Further, we confirmed that OGD-induced autophagy was associated with the activation of cathepsin B and L–tBid–mitochondrial apoptotic signaling pathway using Atg5−/− and Atg5+/+ mouse embryo fibroblast cells. Knockout of *Atg5* inhibited OGD-induced increase of active cathepsin B (Supplementary Figures S4c and k) and L (Supplementary Figures S4d and l) at 6 or 3 h post-OGD, OGD-induced increase of tBid (Supplementary Figures S4e and m), cytoplastic Cyt-c (Supplementary Figures S4g and o), and active caspase-3 (Supplementary Figures S4h and p), and OGD-induced reduction of mitochondrial Cyt-c (Supplementary Figures S4f and n) at 12 h post-OGD.

### Inhibition of autophagy reduces OGD-mediated release of cathepsin B and L from the lysosome into the cytoplasm and activation of caspase-3 in astrocytes

**Figure 4:**
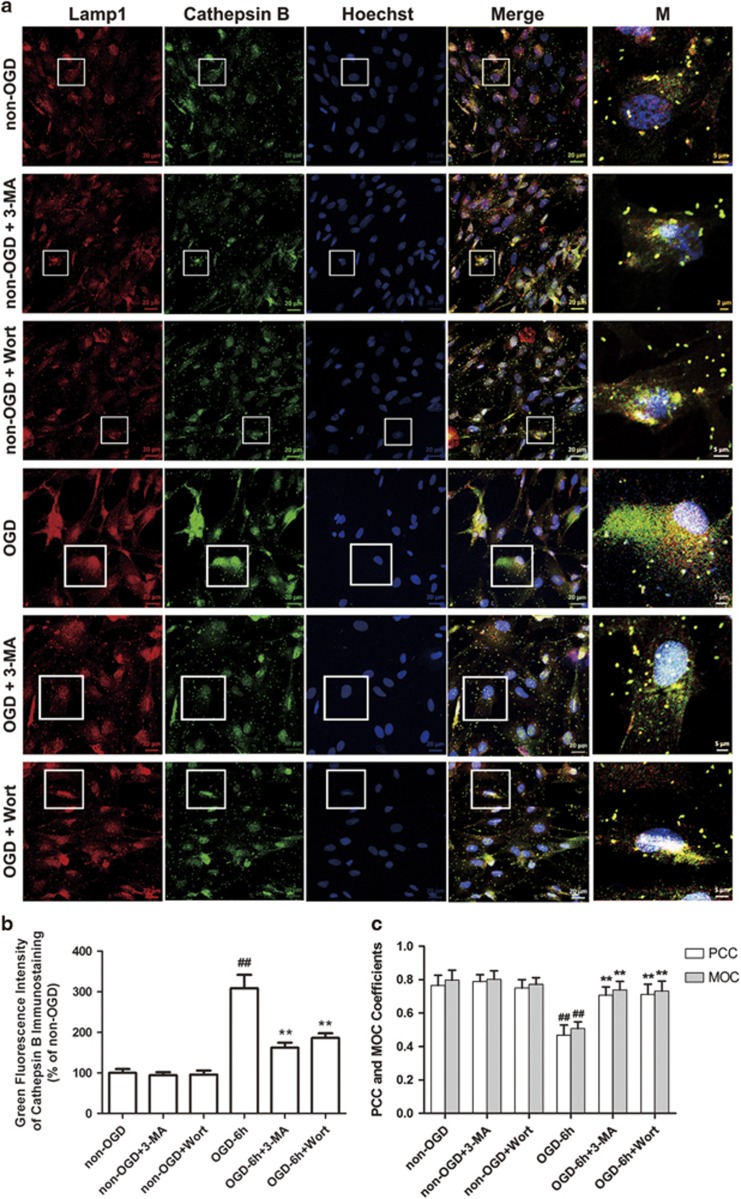
Inhibition of autophagy attenuates OGD-induced release of cathepsin B in astrocytes. (**a**) Representative immunofluorescence staining images of cathepsin B and Lamp 1 in astrocytes. The cells were treated with OGD for 6 h, and 3-MA (1 mM) or Wort (100 nM) was added in the cells 30 min or 2 h before OGD, respectively. Then double immunofluorescence staining of cathepsin B (green) and Lamp 1 (red) in astrocytes was performed by corresponding antibodies. Hoechst (blue) was used to stain nuclei. Images were captured by a confocal microscopy. Magnified images (M) were cropped sections from the merge images (white borders). (**b**) Quantification of green fluorescence intensity of cathepsin B immunostaining in (**a**). (**c**) Pearson's correlation coefficient (PCC) and Manders' overlap coefficient (MOC) demonstrated the colocalization between cathepsin B and Lamp 1. Image-Pro Plus was used to calculate the colocalization coefficients. Means±S.D., *n*=6. ^##^*P*<0.01 *versus* non-OGD group; **P<0.01 *versus* OGD group

**Figure 5:**
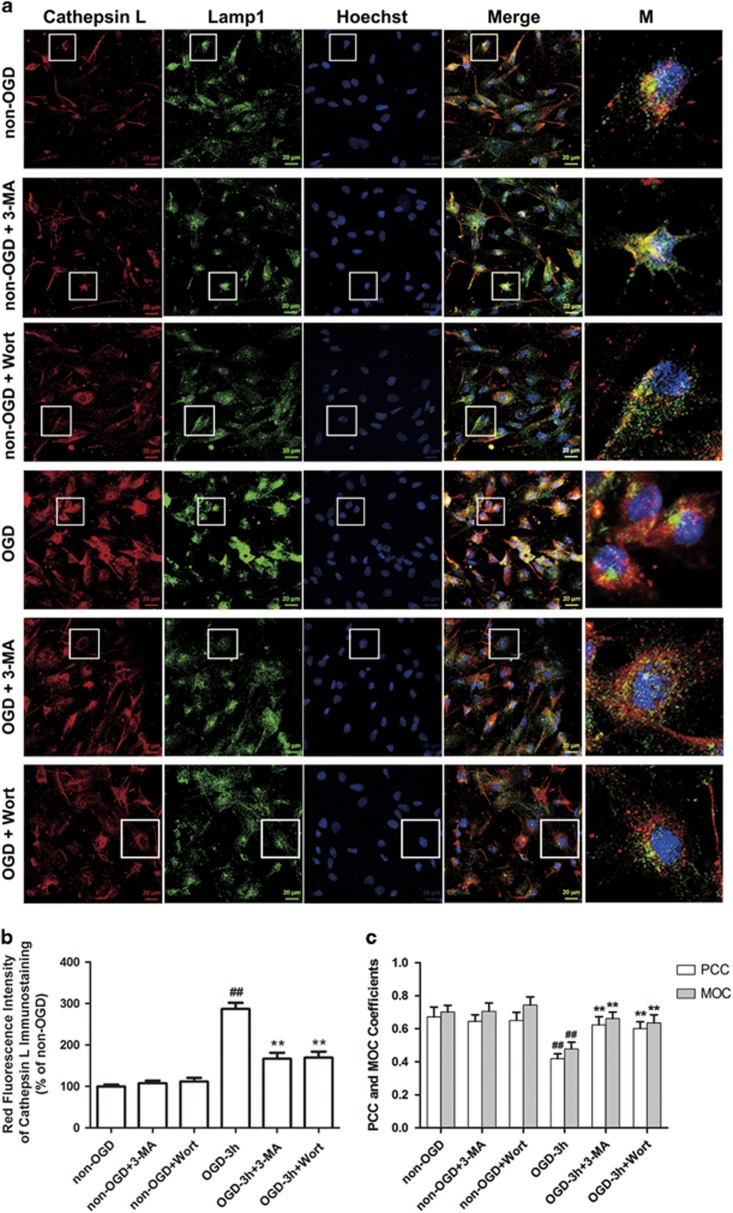
Inhibition of autophagy reduces OGD-induced release of cathepsin L in astrocytes. (**a**) The cells were treated with OGD for 3 h, and 3-MA (1 mM) or Wort (100 nM) was added in cells 30 min or 2 h before OGD, respectively. Then double immunofluorescence staining of cathepsin L (red) and Lamp 1 (green) was performed by corresponding antibodies. Hoechst (blue) was used to stain nuclei. Images were captured by a confocal microscopy. Magnified images (M) were cropped sections from the merge images (white borders). (**b**) Quantification of red fluorescence intensity of cathepsin L immunostaining in (**a**). (**c**) PCC and MOC demonstrated colocalization between cathepsin L and Lamp 1. Image-Pro Plus was used to calculate the colocalization coefficients. Means±S.D., *n*=6. ^##^*P*<0.01 *versus* non-OGD group; **P<0.01 *versus* OGD group

We next tested the effects of 3-MA or Wort on the release of cathepsin B and L from the lysosome into the cytoplasm and the activation of caspase-3 induced by OGD in astrocytes with immunofluorescence. As shown in Figures 4 and 5, there were less fine, granular, perinuclear cathepsin B (Figure 4) and L (Figure 5) immunostaining, which colocalized with Lamp 1-positive lysosomes in non-OGD astrocytes. This finding was consistent with the predominantly lysosomal location of these proteases.^15, 24, 32, 33, 34^ In astrocytes treated with OGD, cathepsin B and L granules became larger and irregular, formed aggregates or showed diffuse cytoplasmic staining at 6 h (Figure 4) or 3 h (Figure 5), and only partially colocalized with the Lamp 1-positive lysosomes, indicating an increased leakage of these two enzymes from the lysosomes into the cytoplasm. In contrast, 3-MA or Wort treatment reduced the aggregates or diffusion of cathepsin B at 6 h (Figure 4) or cathepsin L at 3 h (Figure 5) post-OGD.

**Figure 6:**
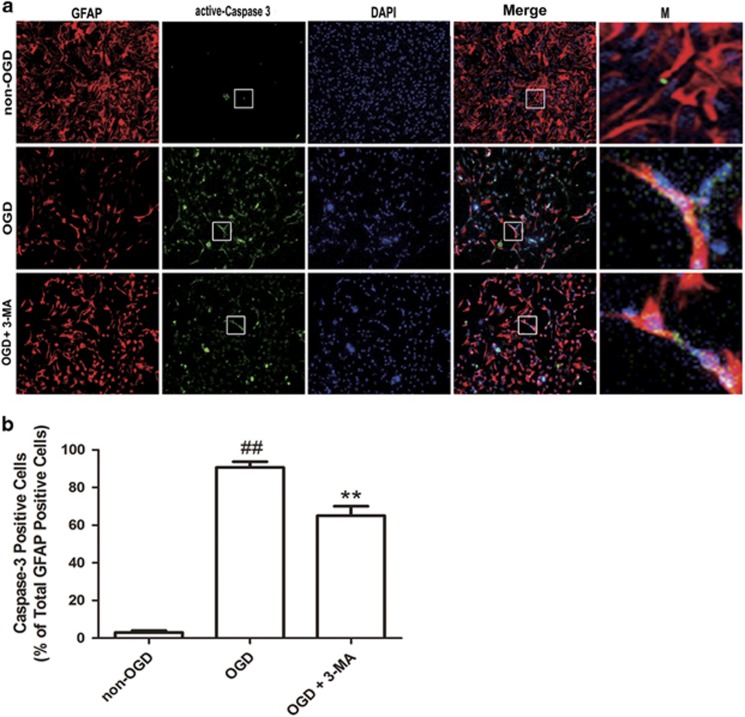
The treatment of 3-MA inhibits OGD-induced activation of caspase-3 in astrocytes. (**a**) Astrocytes were treated with 3-MA (1 mM) and underwent OGD treatment for 12 h, and then the double immunofluorescence staining of caspase-3 (green) and GFAP (red) in astrocytes was performed by corresponding antibodies. DAPI (blue) was used to stain nuclei. Images were captured by the confocal microscopy. Magnified images (M) were cropped sections from the merge images (white borders). Magnification × 200. (**b**) Quantification of active capase-3-positive cells as a percentage of total GFAP-positive cells. Means±S.D., *n*=3. ^##^*P*<0.01 *versus* non-OGD group; **P<0.01 *versus* OGD group

We further tested the effects of 3-MA on OGD-induced activation of caspase-3 in astrocytes with immunostaining. The results showed that much less active caspase-3 immunoreactivity was seen in non-OGD astrocytes (Supplementary Figure S5). In astrocytes treated with OGD, the active caspase-3-positive astrocytes increased over time and peaked at 12 h after OGD (Supplementary Figure S5). In contrast, 3-MA reduced active caspase-3-positive astrocytes at 12 h after OGD (Figure 6).

In addition, we confirmed the role of caspase-3, z-VAD-fmk (nonspecific caspase inhibitor) and Q-DEVD-OPh (a specific inhibitor of caspase-3) both reduced the protein levels of caspase-3 (Supplementary Figures S6a and c, b and d), suggesting that caspase-3 is activated in our OGD model system. To further confirm the role of caspase-3, the LDH leakage was measured. Both z-VAD-fmk and Q-DEVD-OPh at 25 and 50 *μ*M markedly decreased the leakage of LDH in astrocytes 12 h post-OGD (Supplementary Figures S6e and g, f and h), indicating that inhibition of caspases or caspase-3 has a protective effects on ischemic astrocytes. These data further suggest that the protective effects of autophagy inhibition on ischemic astrocytes are potentially mediated by inhibiting the activation of caspase-3.

### Inhibition of autophagy decreases OGD-induced LMP in astrocytes

**Figure 7:**
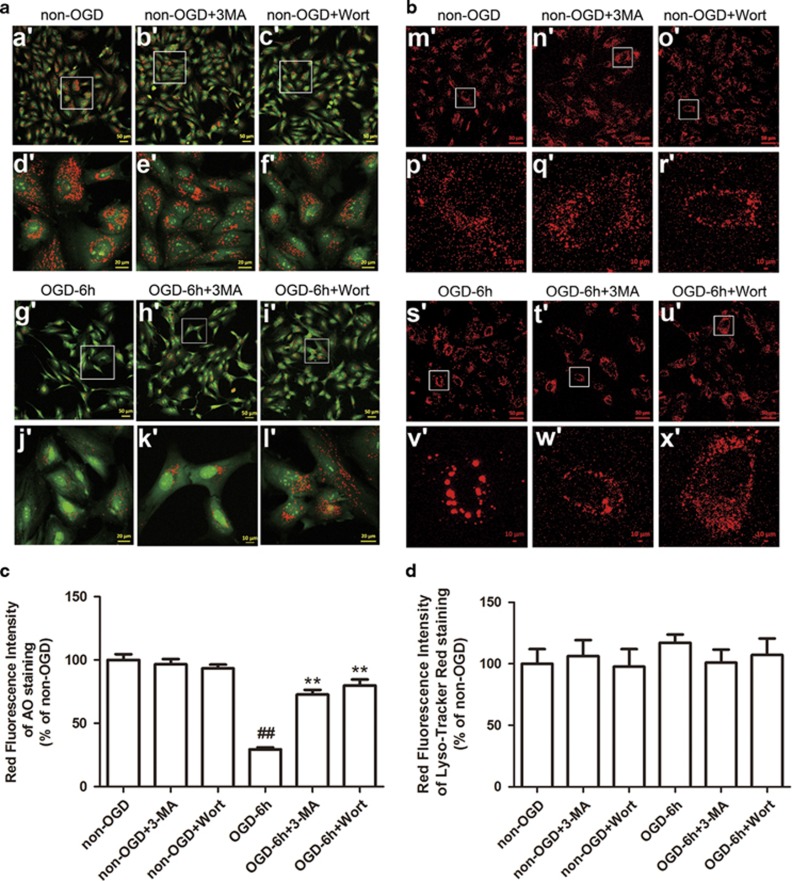
Inhibition of autophagy decreases LMP in OGD-treated astrocytes with AO-uptake and Lyso-Tracker Red uptake methods. (**a** and **b**) Representative photomicrographs of AO staining (**a**) or Lyso-Tracker Red staining (**b**). Cells were treated with OGD for 6 h, and then incubated with AO (5 *μ*g/ml) for 15 min or Lyso-Tracker Red (75 nM) for 60 min. 3-MA (1 mM) or Wort (100 nM) was added in cells 30 min or 2 h before OGD, respectively. The pictures were captured by a confocal microscope. Magnified images (d', e', f', j', k', l', p', q', r', v', w', x') were cropped sections from the white borders areas in the images (a', b', c', g', h', i', m', n', o', s', t', u'), respectively. (**c** and **d**) Quantification of red fluorescence intensity of AO staining (**c**) or Lyso-Tracker Red staining (**d**). Means±S.D., *n*=6.^**##**^*P*<0.01 *versus* non-OGD group; **P<0.01 *versus* OGD group

Excessive autophagy induces LMP^35, 36^ and it is possible that LMP mediates cathepsin B and L cytosolic translocation. Hence, we evaluated LMP formation by Acridine Orange (AO) and Lyso-Tracker Red staining assays. Normally, AO, a metachromatic fluorophore cloistering inside of the lysosome, exhibits a high level of red fluorescence and a low level of green fluorescence. When lysosomes are disrupted, AO relocates to the cytosol from the lysosomes and manifests a reduced red fluorescence and an increased green fluorescence.^36^ As shown in Figures 7a and c, OGD induced a reduction in red fluorescence in astrocytes. In contrast, treatment with 3-MA or Wort markedly inhibited OGD-induced reduction in red granular fluorescence of AO staining. Lyso-Tracker Red uptake images in astrocytes further indicated that 3-MA or Wort treatment attenuated OGD-induced lysosomal destabilization manifested by a reduction in lysosome swelling and rupture (Figures 7b and d). The above data suggest that 3-MA or Wort can stabilize OGD-induced lysosomal membrane instability in astrocytes.

### Inhibition of autophagy enhances OGD-induced upregulation in lysosomal heat shock protein 70.1B (Hsp70.1B) in astrocytes

**Figure 8:**
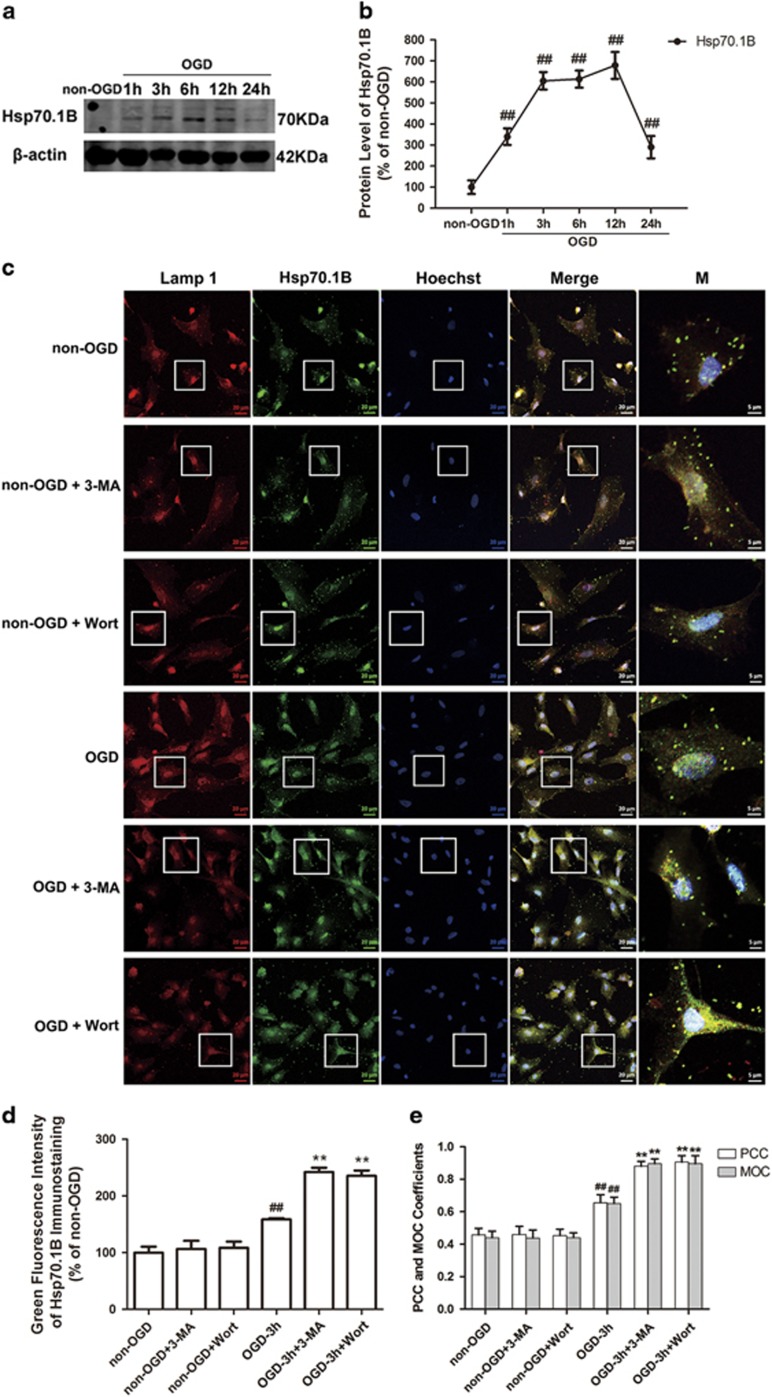
Inhibition of autophagy further increases OGD-induced upregulation of Hsp70.1B in astrocytes. (**a**) Representative western blotting analysis for the protein levels of Hsp70.1B at different time-points after OGD treatment. (**b**) The line represents quantitative analysis of immunoblots in (**a**). Means±S.D., *n*=3. ^**##**^*P*<0.01 *versus* non-OGD group. (**c**) The cells were treated with OGD for 3 h. 3-MA (1 mM) or Wort (100 nM) was added in the cells 30 min or 2 h before OGD, respectively. Then double immunofluorescence staining of Lamp 1 (red) and Hsp70.1B (green) was performed by corresponding antibodies. Hoechst (blue) was used to stain nuclei. Images were captured by a confocal microscopy. Magnified images (M) were cropped sections from the merge images (white borders). (**d**) Quantification of green fluorescence intensity of Hsp70.1B immunostaining in (**c**). (**e**) PCC and MOC demonstrated the colocalization between Hsp70.1B and Lamp 1. Image-Pro Plus was used to calculate colocalization coefficients. Means±S.D., *n*=6. ^**##**^*P*<0.01 *versus* non-OGD group; **P<0.01 *versus* OGD group

Hsp70.1B is known to stabilize lysosomal membrane by recycling damaged proteins and protect cells from various insults such as heat, ischemia and other oxidative stresses.^37, 38, 39^ The chaperone function and inhibition of lysosomal membranes permeabilization or rupture are the major mechanisms by which Hsp70.1B protects cells.^39, 40, 41^ We found that OGD induced a significant increase in Hsp70.1B level during the period of 3–12 h post-OGD in astrocytes (Figures 8a and b). Double immunofluorescence staining of Hsp70.1B and Lamp 1 showed that in non-OGD astrocytes, there was less immunoreactive colocalization of Hsp70.1B with Lamp 1 (Figures 8c–e). After OGD, the immunoreactivities of Hsp70.1B became apparent, and upregulated Hsp70.1B was colocalized with Lamp 1, indicating the translocation of Hsp70.1B to the lysosomal membrane (Figures 8c–e). Surprisingly, Hsp70.1B colocalized with Lamp 1 was more intense when 3-MA or Wort was added to the astrocytes (Figures 8c–e). These data indicate that the inhibition of autophagy upregulates the lysosomal Hsp70.1B, possibly contributing to a reduction in OGD-induced lysosomal membrane instability in astrocytes.

## Discussion

To date, it is well accepted that autophagy is a major mediator of neuronal cell death in cerebral ischemia.^9, 10, 11, 28, 42, 43^ In 2010, we first reported that autophagy is activated in ischemic astrocytes and contributes to astrocytic cell death.^12^ Similarly, Pamenter *et al.*^44^ found that astrocytes are more sensitive to conditions mimicking metabolic and ischemic stress of penumbral tissue than neurons and exhibit a stronger autophagic response to these stresses. Recent advances have elucidated that autophagy and apoptosis can share common regulators,^45, 46, 47, 48^ such as Bcl-2, which has been identified as a central regulator of autophagy and apoptosis by interacting with both Beclin-1 and Bax/Bak, respectively. Several apoptotic proteins (e.g., PUMA, Noxa, Nix, Bax, XIAP and Bim) are also believed to be regulators of autophagy.^48^ However, the molecular mechanisms linking autophagy and apoptosis are not fully defined, especially in ischemic astrocytes. The novel aspect of the present work is that the inhibition of autophagy blocks the activation and release of cathepsin, and lead to the inhibition of tBid–mitochondrial apoptotic signaling pathway involving stabilization of the lysosomal membrane via upregulation of the lysosomal Hsp70.1B in ischemic astrocytes.

The inhibition of autophagy blocks cathepsins–tBid–mitochondrial apoptotic signaling pathway in ischemic cortex. Lysosomal proteases, such as cathepsin B, have important roles in apoptosis via cleavage of Bid, release of Cyt-c and activation of caspases in both neurons and non-neural cells.^15, 16^ Our prior studies demonstrated that cathepsin B and L are activated in the ischemic cortex after pMCAO, and lead to the activation of tBid–mitochondrial apoptotic signaling pathway.^24^ The peak for cathepsin B or L activation was at 6 or 3 h post-ischemia, respectively. The maximal increase in tBid, cytoplastic Cyt-c and active caspase-3 and the maximal reduction in mitochondrial Cyt-c were at 12–24 h post-ischemia. Our present data and others showed that 3-MA treatment at 300–600 nmol (icv) reduced infarct volume and improved neurological deficits in rat models of pMCAO.^11, 12^ Our previous study also found that 3-MA treatment at 300–600 nmol (icv) could protect astrocytes in the ischemic cortex.^12^ In the current studies, we further found that 3-MA treatment at 300–600 nmol (icv) could inhibit ischemia-induced increase in active cathepsin B or cathepsin L at 6 or 3 h post-ischemia, reverse ischemia-mediated increase in tBid, cytoplastic Cyt-c and active caspase-3, and ischemia-mediated reduction in mitochondrial Cyt-c at 24 h after ischemia. These data indicate that the ischemia-induced autophagy activation confers the activation of cathepsin B and L, the cleavage of Bid, the translocation of Cyt-c from the mitochondria to the cytosol and the activation of caspase-3 in the ischemic cortex.

The inhibition of autophagy blocks cathepsins–tBid–mitochondrial apoptotic signaling pathway in ischemic astrocytes. Previous studies demonstrated that a higher dose of 3-MA (10 mM) could inhibit TNF-induced autophagy in FADD-deficient Jurkat cells,^31^ and pre-treatment with 3-MA (10 mM) reduced staurosporine-induced neuronal death.^49^ In the previous study, we also found a higher dose of 3-MA (10 mM) exhibits a mild protection against OGD-induced astrocytes injury. In the current study, we further demonstrated that low doses of 3-MA (0.1, 0.5, or 1 mM) or Wort also protected astrocytes against OGD-induced injury.

Previously, we reported that OGD induces an increase in activated cathepsin B and cathepsin L, tBid, activated caspase-3, and cytoplastic Cyt-c and a reduction in mitochondrial Cyt-c in astrocytes at 3–12 h post-OGD. Inhibition of cathepsin B or L confers protective effect on ischemic astrocytes via inhibiting the activation of tBid–mitochondrial apoptotic signaling pathway. In the current study, we further found that the pharmacological or genetic inhibition of autophagy reversed OGD-induced increase in active cathepsin B and L, tBid, active caspase-3 and cytoplastic Cyt-c and OGD-induced reduction in mitochondrial Cyt-c in astrocytes. Our above data suggest that the activation of autophagy in the ischemic astrocytes may be involved in apoptotic regulation via activating lysosomal proteases, leading to the cleavage of Bid, the release of the mitochondrial Cyt-c into the cytosol and the activation of caspase cascade. Atg5 is an autophagy-specific gene required for autophagosome precursor synthesis and its deletion in yeast, mammalian cells and mice effectively blocks autophagy.^50^ In support of this finding, knockout of *atg5* also protected OGD-induced mouse embryo fibroblast cells injury and inhibited OGD-induced activation of cathepsin B or cathepsin L–tBid–mitochondrial apoptotic signaling pathway.

The inhibition of autophagy blocks OGD-induced translocation of cathepsin B/L from the lysosome into the cytoplasm and the activation of caspase-3 in astrocytes. Along with others, we have found that cathepsin B or L is normally confined to the endolysosomal compartment in neuron and astrocyte. When ischemia occurs, cathepsin B or L translocates to the cytoplasm from the lysosome, and leads to the activation of tBid–mitochondrial apoptotic signaling pathway.^24, 51^ One of the novel finding of this study is that 3-MA or Wort reversed OGD-induced release of cathepsin B or cathepsin L from the lysosomes into the cytoplasm and the activation of caspase-3 in astrocytes. In addition, we confirmed that caspase-3 plays a role in ischemic astrocytic injury associating with autophagy activation in our model system.

The inhibition of autophagy decreases OGD-induced LMP in astrocytes. The movement of lysosomal cathepsin B or L into the cytosol can be used to measure the LMP in neurons or in astrocytes.^24, 29^ Excessive autophagy leads to LMP induction.^35, 36^ Another novel finding of this study is that the inhibition of autophagy by 3-MA or Wort can stabilize the OGD-induced lysosomal membrane instability in astrocytes.

The inhibition of autophagy enhances OGD-induced upregulation of lysosomal Hsp70.1B in astrocytes. Hsp70.1 is one major protein of human Hsp70 family, and mainly functions as a chaperone enabling the cell to deal with harmful aggregations of denatured proteins upon various insults such as heat, ischemia and other oxidative stresses.^37, 38, 39^ In 2010, Sahara *et al.*^39^ demonstrated that Hsp70.1 was upregulated at the lysosomal membranes of neuronal cells after ischemia–reperfusion injury and inhibited LMP. An important unexpected finding of this study is that the inhibition of autophagy by 3-MA or Wort enhanced OGD-induced upregulation of lysosomal Hsp70.1B, perhaps contributing to a reduction in OGD-induced lysosomal membrane instability in astrocytes. This finding confirmed the link between Hsp70.1 and autophagy, which was reported by Sisti.^52^ However, the molecular mechanisms underlying the upregulation of lysosomal Hsp70.1B by 3-MA or Wort requires further investigation.

In conclusion, the current study provides the first evidence that inhibition of autophagy blocks activation and release of cathepsins via stabilization of lysosomal membrane. This effect may result from upregulation of lysosomal Hsp70.1B, leading to inhibition of the tBid–mitochondrial apoptotic signaling pathway in ischemic astrocytes.

## Materials and methods

### Animals

Male Sprague-Dawley rats weighing 280–320 g were purchased from the Center for Laboratory Animals, Soochow University,Suzhou, China (production license: XCYK- 2002-0008). Animal procedures were performed according to a protocol approved by the Institutional Animal Care and Use Committee of Soochow University, Suzhou, China.

### pMCAO model

pMCAO model was prepared as described previously.^12^ Briefly, rats were anesthetized with intraperitoneal injection of 4% choral hydrate (350 mg/kg). Permanent focal cerebral ischemia was induced by a 30 mm length of 4-0 nylon monofilament suture (Φ 0.22–0.24 mm) inserted from the right common carotid artery (CCA) to the internal carotid artery through a small incision in the CCA, and then advanced to the circle of Willis to occlude the origin of the right middle cerebral artery. Body temperature was maintained at 37 °C by a heating pad during and after surgery until recovery from anesthesia. Sham-operated rats underwent the same procedures except for inserting a nylon monofilament suture to the artery. 3-MA (08592, Sigma-Aldrich, St. Louis, MO, USA) or vehicle was administrated icv 10 min after ischemia.

### Primary cortical astrocyte culture

Primary astrocyte culture was performed as previously described.^12^ Briefly, dissected cerebral cortexes from Sprague–Dawley neonates (1- or 2-day-old) were digested with 0.25% trypsin for 10 min at 37 °C, and filtered through a sterile 40 *μ*m nylon cell strainer. Astrocytes were suspended in DMEM/F12(1:1) (GIBCO, Thermo Fisher Scientific,Waltham, MA, USA, 11330) containing 10% heat-inactivated fetal bovine serum (GIBCO, 10099) and 1% 100 U/ml penicillin/streptomycin (Beyotime, Jiangsu, China, C0222), and seeded onto dishes or plates coated with poly-l-lysine, and then incubated under a humidified atmosphere with 5% CO_2_ at 37 °C. We used astrocytic marker protein GFAP to detect the purity of astrocytes by immunocytochemistry, showing a satisfactory result that >95% of the cells were GFAP positive.

### Oxygen and glucose deprivation

For OGD treatment, cells were rinsed twice with phosphate-buffered saline and refreshed with glucose-free DMEM (GIBCO, 11966), and placed in a sealed chamber for indicated period (Billups-Rothenberg, San Diego, CA, USA) that was continuously flushed with mixed gas containing 95% N_2_ and 5% CO_2_ for 10 min. The control cells were incubated in glucose-containing DMEM in a humidified atmosphere with 5% CO_2_ at 37 °C. 3-MA (08592, Sigma) at 0.1, 0.5 and 1 mM, Wort (W3144, Sigma) at 25, 50 and 100 nM, z-VAD (ab120382, Abcam, Cambridge, UK) at 25, 50 and 100 *μ*M or Q-DEVD-OPh (ab142037, Abcam) at 25, 50 and 100 *μ*M was diluted with complete medium at different concentrations, and added to cells 30 min, 2 h, 1 h or 30 min before OGD treatment, respectively.

### Lentiviruses transfection

The lentiviruses with short hairspin RNA targeting *atg5* (shRNA Atg5) and control scrambled shRNA (scr shRNA) were produced by GeneChem Co., Ltd (Shanghai, China). The target sequence for *atg5* as follows: shRNA Atg5: 5′-TGAGATAACTGAACGAGAA-3′; scr shRNA: 5′-TTCTCCGAACGTGTCACGT-3′. Lentiviruses were added to the third generation of primary cultured astrocytes and transfected for 72 h. The transfection efficiency was >80% (data not shown). Western blotting analysis confirmed that the *atg5* gene was successfully silenced in astrocytes (Supplementary Figure S2a and g).

### Western blotting analysis

The collected cortical tissue or cells was added in lysing buffer with protease inhibitor cocktail (Roche, Basel, Schweiz, 04693159001) and sonicated on ice. Protein concentrations were determined by a BCA protein assay kit (Pierce, Rockford, IL, USA). The proteins were separated using SDS-PAGE and transferred to a nitrocellulose membrane, and then blocked with 5% non-fat milk for 1 h. Blots were incubated with specific primary antibodies overnight at 4 °C and corresponding secondary antibodies for 1 h at room temperature. Blots were captured by odyssey scanner (LI-COR, Bioscience, Lincoln, NE, USA). Densitometric analysis of the bands is quantitatively analyzed with Sigma Scan Pro 5 (Sigma-Aldrich, St. Louis, MO, USA). The antibodies used in this study are listed in the Supplementary Tables S1 and S2.

### Immunofluorescence

Cells planted on 24-well plates were fixed with 4% paraformaldehyde for 5 min, permeabilized and blocked with 1% BSA containing 0.1% Triton X-100 for 1 h at room temperature, and incubated overnight at 4 °C in specific primary antibodies. The cells were subsequently incubated (1 h, room temperature) with corresponding secondary antibodies. Then, the cells were incubated with DAPI (1 : 10 000, D9564, Sigma) or Hoechst (1 : 10 000, 33258, Sigma) solution for 10 or 30 min, respectively, to stain nuclei. Images were obtained by fluorescence or confocal microscope. The antibodies used in this study are listed in the Supplementary Tables S1 and S2.

### Measurement of lysosomal stability

AO (318337, Sigma-Aldrich) and Lyso-Tracker Red (C1046, Beyotime) staining assays were widely used for evaluating the LMP.^36, 53, 54^ AO is a lysosomotropic base and a metachromatic fluorophore. Normally, AO captures protons inside the acidic vacuolar compartment, preferentially in secondary lysosomes, and retains its charged form, which results in red fluorescence. When LMP is increased, AO relocates to the cytosol from the lysosomes, leading to cytoplasmic diffuse green fluorescence and reduced red fluorescence. Cells were suffered OGD treatment for 6 h, and then incubated with 5 *μ*g/ml of AO in complete medium for 15 min at 37 °C, or stained with Lyso-Tracker Red (75 nM) in the dark for 60 min at 37 °C. 3-MA (1 mM) or Wort (100 nM) was added in medium 30 min or 2 h before OGD, respectively. Images were acquired using a confocal laser scanning microscopy (LSM 710, Carl Zeiss, Oberkochen, Germany).

### Statistical analysis

Data are expressed as mean±S.D., statistical analysis was carried out by one-way ANOVA followed by the Tukey's post-hoc test with Prism software (La Jolla,CA, USA). Significant difference was set at *P*<0.05. Image-Pro Plus (Rockville, MD, USA) was used to calculate the colocalization coefficients.

Other Materials and Methods are available in the Supplementary Materials and Methods.
