# Supplementary material for: Inhibition of autophagy blocks cathepsins–tBid–mitochondrial apoptotic signaling pathway via stabilization of lysosomal membrane in ischemic astrocytes
Source: Cell Death Dis. 2017 Feb 16;8(2):e2618–. doi: 10.1038/cddis.2017.34 (PMC5386481; doi:10.1038/cddis.2017.34)
Supplement: Supplementary Material [file cddis201734x1.docx]

**Inhibition of autophagy blocks cathepsins-tBid-mitochondrial apoptotic signaling pathway via stabilization of lysosomal membrane in ischemic astrocytes**

Xian-Yong Zhou ^1,†^, Yu Luo^1, †^, Yong-Ming Zhu^1, †^, Zhi-He Liu^2^, Thomas A. Kent^3^, Jia-Guo Rong^1^, Wei Li^1^, Shi-Gang Qiao^1^, Min Li^1^, Yong Ni^1^, Kazumi Ishidoh^4^ and Hui-Ling Zhang^1,*^

^1^Jiangsu Key Laboratory of Translational Research and Therapy for Neuro-Psycho-Diseases, College of Pharmaceutical Science; Department of Pharmacology and Laboratory of Cerebrovascular Pharmacology; Jiangsu Key Laboratory of Preventive and Translational Medicine for Geriatric Diseases, School of Public Health, Soochow University, Suzhou, 215123, China

^2^Guangzhou Institute of Traumatic surgery, Guangzhou Red Cross Hospital, Medical

College, Jinan University, Guangzhou 510220, China

^3^Stroke Outcomes Laboratory, Department of Neurology, Baylor College of Medicine, Houston, TX; and Center for Translational Research on Inflammatory Diseases, Michael E. DeBakey Veterans Affairs Medical Center, Houston 77030, TX

^4^Institute for Health Sciences, Tokushima Bumi University, 180 Nishihamabouji, Yamashiro-cho, Tokushima City, Tokushima 770-8514, Japan

**Supplementary Material**

**Materials and Methods**

**Mouse embryo fibroblasts (MEFs) culture.** Atg5-/- MEFs and wide type (WT) MEFs were kindly provided by Professor Guanghui Wang, the Department of Pharmacology, Soochow University. Atg5-/- MEFs and WT MEFs were cultured in Dulbecco's Modified Eagle's Medium (DMEM)(Sigma, D5796) supplemented with 10% heat-inactivated fetal bovine serum (GIBCO, 10099) and 1% 100 U/ml penicillin/streptomycin (Beyotime, C0222) under a humidified atmosphere with 5% CO2 at 37°C.

**LDH leakage measurement.** Cell injury was evaluated by assaying lactate dehydrogenase (LDH) level in cultured medium. A LDH assay kit (Nanjing Jiancheng Bioengineering Institute, Nanjing, PR China) was used to measure LDH level at 450 nm with an automatic multiwell spectrophotometer (Bio-Rad Laboratories, Hercules, CA, USA), according to the manufacturer’s instructions.

**Extract the mitochondria and cytoplasm.** Isolation of the mitochondria and cytoplasm was performed as described previously.^25^ The cortical tissue or cells was homogenized in a specified amount of buffer A (250 mM sucrose, 1 mM EDTA, 50 Mm Tris-HCl, 1 mM dithiothreitol) with protease inhibitor cocktail (Roche, 04693159001), and centrifuged at 1000 g for 10 min at 4°C, and then the resultant supernatant was centrifuged at 10,000 g for 20 min at 4°C to acquire the supernatant and mitochondria precipitation. Then, the supernatant was transferred to a new tube immediately and centrifuged at 100,000 g for 60 min at 4°C to extract the cytosolic fraction. The mitochondrial precipitation was washed three times in buffer B (250 mM sucrose, 1 mM EGTA, 10 mM Tris-HCl ), and then centrifuged at 10,000 g at 4°C for 10 min to obtain the pure mitochondria. The protein concentration of pure mitochondria and cytoplasm were determined by BCA protein assay kit (Pierce, Rockford, IL, USA) and the protein levels of mitochondrial and cytoplastic Cyt-c were detected by western blotting analysis.

**.**

**Supplementary Figure**

Supplementary Figure S1


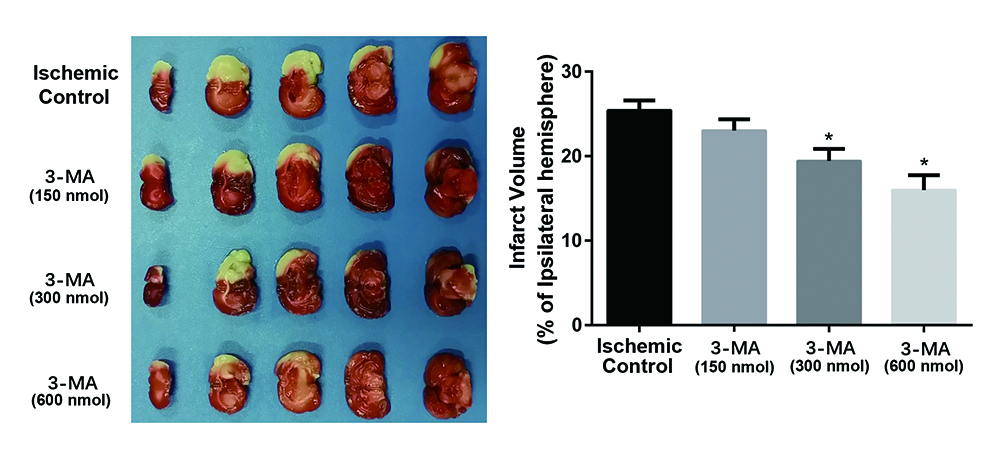


**Supplementary Figure S1**. 3-MA treatment reduces brain infarct volume induced by pMCAO. 3-MA (150, 300, 600 nmol) or vehicle was administrated intracerebroventricularly (icv) 10 min after ischemia induced by pMCAO. The brains were sliced and stained with TTC. The white area represents the infarct brain tissue. Columns represent quantitative analysis of brain infarct volume. Statistical analysis was performed with one-way ANOVA followed by a post hoc Tukey test. Means ± SD, n=10. *P < 0.05 vs. ischemic control group.

Supplementary Figure S2

**
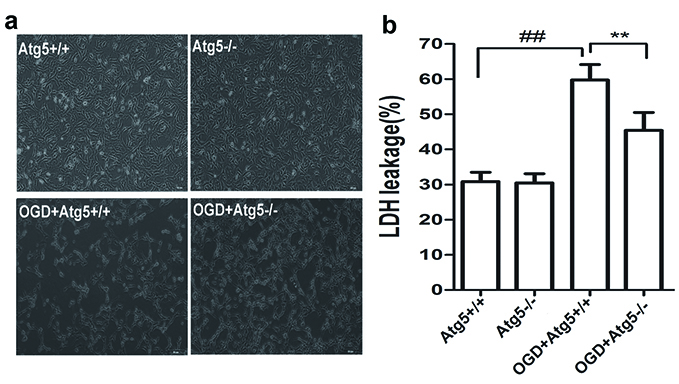
Supplementary Figure S2**. Knockout of *Atg5* protects mouse embryo fibroblast (MEF) cells against oxygen glucose deprivation (OGD) injury. MEF cells were suffered OGD treatment for 12 h. (**a**) Light microscope images showed that knockout of *atg5* significantly improved the morphology of OGD-treated MEF cells. (**b**) LDH leakage analysis showed that knockout of *atg5* decreased the LDH leakage. Means ± SD, n = 6. Statistical analysis was performed with one-way ANOVA followed by a post hoc Tukey test. ^##^P< 0.01 vs. Atg5^+/+^ group; **P < 0.01 vs. OGD+Atg5^+/+^ group.

Supplementary Figure S3
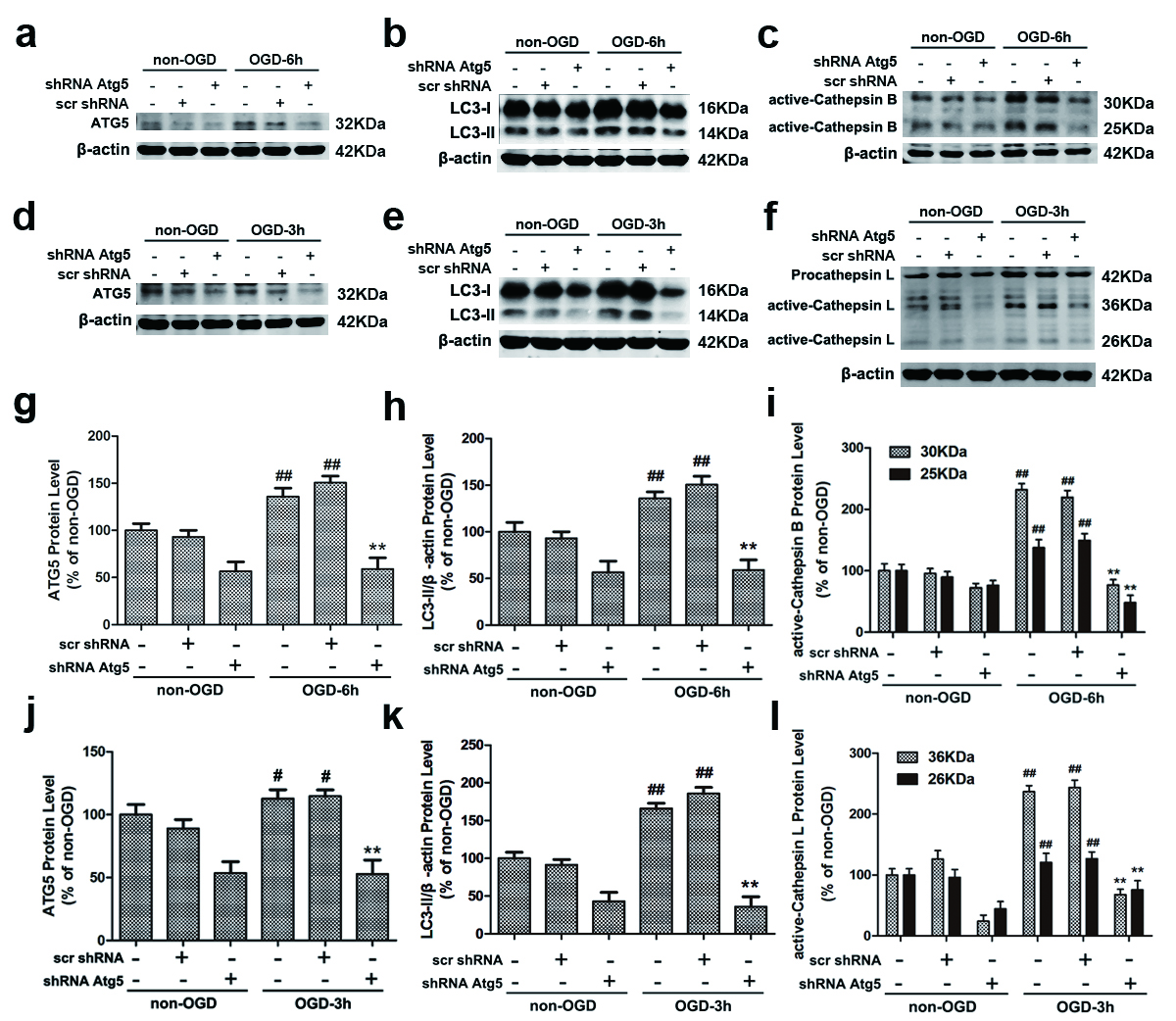


**Supplementary Figure S3.** Knockdown of *Atg5* inhibits OGD-induced activation of cathepsin B or cathepsin L in astrocytes. Lentiviruses with shRNA Atg5 were added to the 3nd generation of primary cultured astrocytes. (**a-f)** Representative western blotting images showed the protein level changes of ATG5 (**a**), LC3-II (**b**), active cathepsin B (**c**) at 6 h or ATG5 (**d**), LC3-II (**e**), active cathepsin L (**f**) at 3 h after OGD. (**g-l**) Columns represent quantitative analysis of immunoblots in **a-f**, respectively (means ± SD, n=3). β-actin was used as a loading control. Statistical analysis was performed with one-way ANOVA followed by a post hoc Tukey test. ^#^P < 0.05, ^##^P< 0.01 vs. non-OGD + scr shRNA group; **P < 0.01 vs .OGD + scr shRNA group.

Supplementary Figure S4


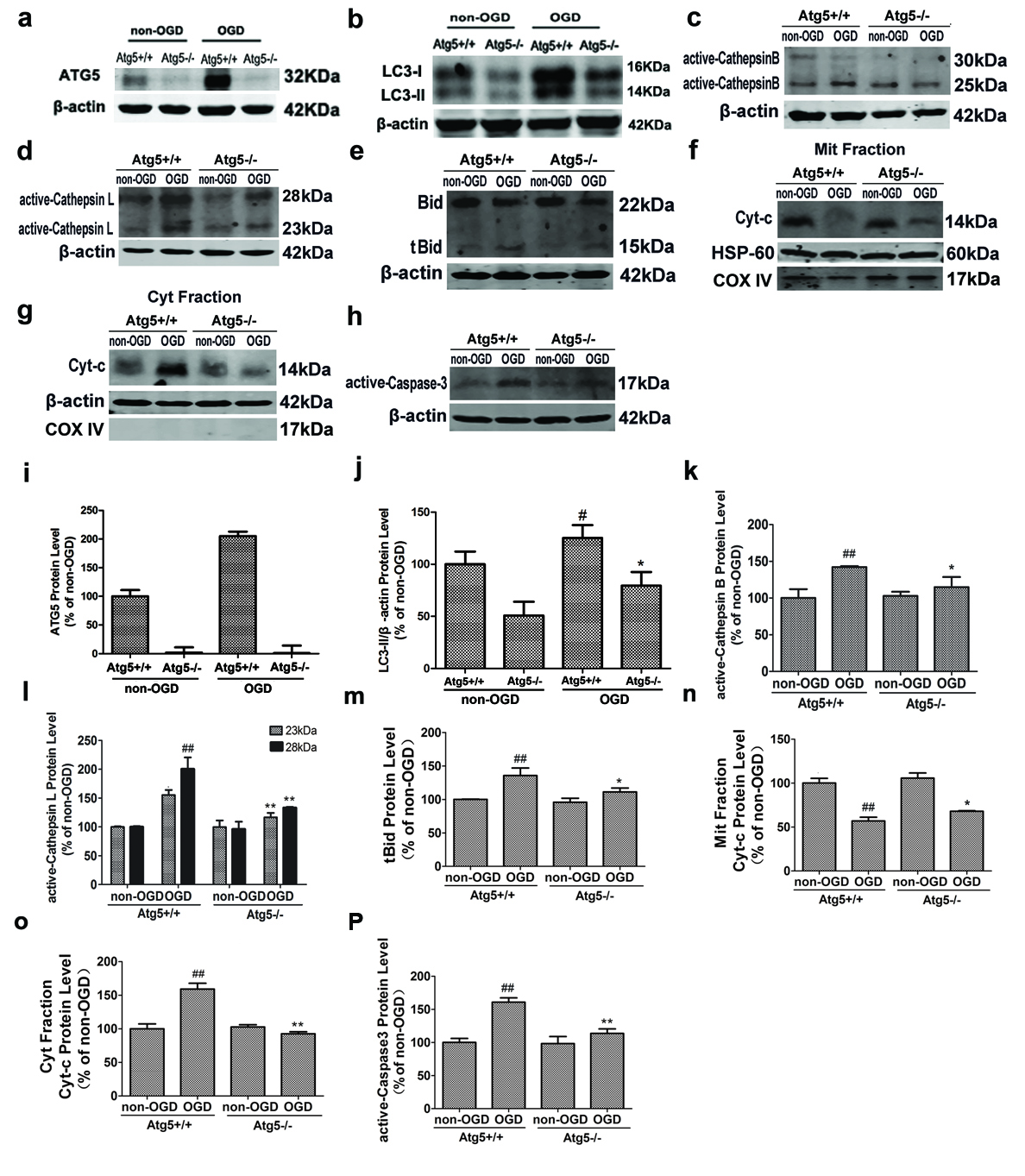


Supplementary Figure S4. Knockout of *Atg5* inhibits OGD-induced activation of cathepsin B or cathepsin L -tBid-mitochondrial apoptotic signaling pathway in mouse embryo fibroblast (MEF) cells. (**a-h)** Representative western blotting images showed the protein level changes of ATG5 (**a**), LC3-II (**b**), active cathepsin B (**c**) at 6 h or cathepsin L (**d**) at 3 h, tBid (**e**), mitochondrial (**f**) and cytoplastic (**g**) Cyt-c and active caspase-3 (**h**) at 12 h after OGD. (**i-p**) Columns represent quantitative analysis of immunoblots in **a-h**, respectively (means ± SD, n=3). Cytochrome C Oxidase IV (COX IV), which is located in the inner mitochondrial membrane, acts as a mitochondrial marker. β-actin or HSP-60 was used as a loading control. Statistical analysis was performed with one-way ANOVA followed by a post hoc Tukey test. ^##^P< 0.01 vs. non-OGD group; *P < 0.05, **P < 0.01 vs .OGD group.

Supplementary Figure S5


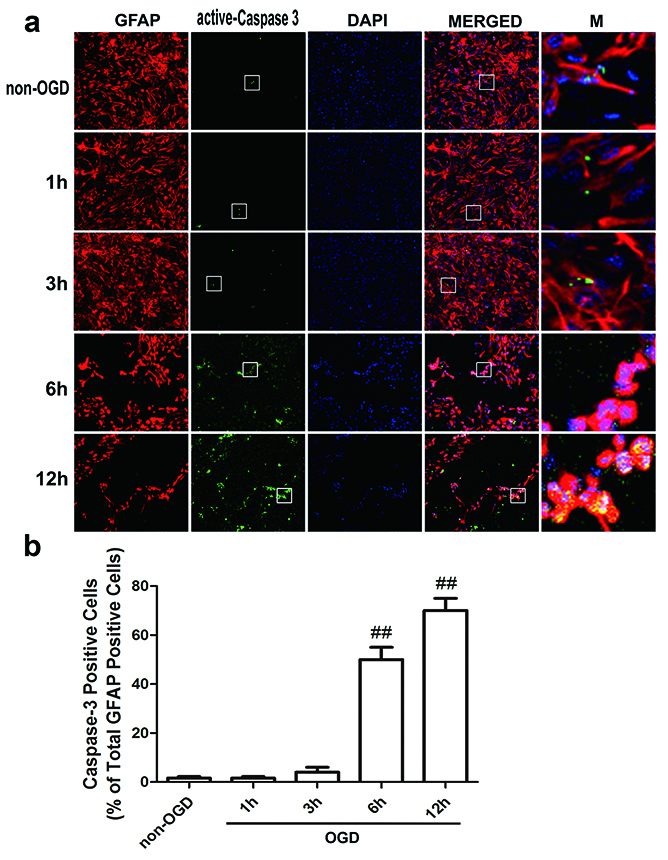


**Supplementary Figure S5**. The time course changes of active capase-3 in OGD-treated astrocytes. (**a**) Astrocytes were suffered OGD treatment for 1h, 3h, 6h and 12h, and the double immunofluorescence staining of caspase-3 (green) and GFAP (red) was performed by corresponding antibodies. DAPI (blue) was used to stain nuclei. Images were captured by the confocal microscopy. Magnified images (M) were cropped sections from the merge images (white borders). Magnification ×200. (**b**) Quantification of active capase-3-positive cells as a percentage of total GFAP-positive cells. Statistical analysis was performed with one-way ANOVA followed by a post hoc Tukey test. Means ± SD, n=3. ^##^P < 0.01 vs. non-OGD group.

Supplementary Figure S6


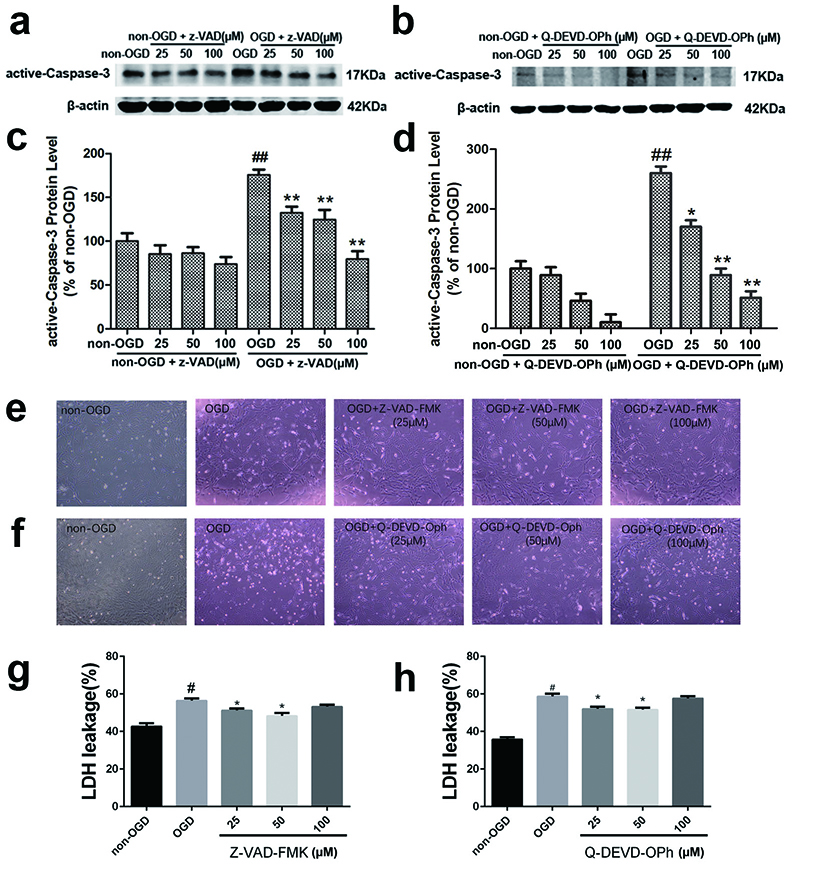


**Supplementary Figure S6**. Inhibition of caspases or caspase-3 has protective effects on ischemic astrocytes. z-VAD-fmk (25, 50, or 100μM) or Q-DEVD-OPh (25, 50, or 100μM) was added in cells 1 h or 30min before OGD, respectively. (**a** and **b**) Representative western blotting images of protein levels of active caspase-3 at 12 h after OGD. (**c** and **d**) Columns represent quantitative analysis of immunoblots in **a** and **b**, respectively (means ± SD, n=3). β-actin was used as a loading control. (**e** and **f**) Representative light microscope images of astrocytes without OGD or with OGD treatments. (**g** and **h**) LDH leakage analysis showed that z-VAD-fmk (25 and 50μM) or Q-DEVD-OPh (25 and 50μM) decreased the LDH leakage of astrocytes with OGD treatment. Means ± SD, n = 6. Statistical analysis was performed with one-way ANOVA followed by a post hoc Tukey test. #P< 0.05 vs. non-OGD group; *P < 0.05 vs. OGD group.

**Supplementary Tables**

**Supplementary Table 1. Primary antibodies used in this study**

| **Protein** | **Usage** | | **Antibody** |
| --- | --- | --- | --- |
| ATG5 | WB (1:500) | PAB13023, Abnova | |
| LC3-Ⅱ | WB (1:400) | M152-3, MBL | |
| Cathepsin L | WB (1:500), IF (1:100) | ab6314, Abcam | |
| Cathepsin B | WB (1:250), IF (1:200) | 06-480, Millipore | |
| Bid | WB (1:500) | AB1735, Millipore | |
| Cyt-c | WB (1:2000) | 2119-1, Epitomics | |
| Caspase-3 | WB (1:250) | AB3623, Millipore | |
| COX IV | WB (1:1000) | AC610, Beyorime | |
| Hsp70.1B | WB (1:300), IF (1:300) | GTX106148, Gene Tex | |
| β-actin | WB (1:5000) | A5441, Sigma | |
| Hsp-60 | WB (1:10000) | 611562, BD Bioscience | |
| Caspase-3 | IF (1:400) | 9661, Cell signaling technology | |
| GFAP | IF (1:500) | C9205, Sigma | |
| GFAP | IF (1:500) | AB5804, Millipore | |
| Lamp1 | IF (1:500) | ab24170, Abcam | |
| Lamp1 | IF (1:100) | ab13523, Abcam | |

Abbreviations: WB, Western blotting; IF, Immunofluorescence.

**Supplementary Table 2. Secondary antibodies used in this study**

| **Protein** | **Usage** | **Antibody** | |
| --- | --- | --- | --- |
| TRITC-labeled goat anti-mouse IgG (H+L) | IF (1:200) | | T5393, Sigma |
| FITC-labeled goat anti-mouse IgG (H+L) | IF (1:200) | | F9006, Sigma |
| FITC-labeled goat anti-rabbit IgG (H+L) | IF (1:200) | | F6005, Sigma |
| Alexa Fluor® 594 goat anti-rabbit IgG (H+L) | IF (1:500) | | A11012, lifetechnologies |
| Alexa Fluor® 594 goat anti-mouse IgG (H+L) | IF (1:500) | | A11005, lifetechnologies |
| Alexa Fluor® 488 goat anti-rabbit IgG (H+L) | IF (1:500) | | A11008, lifetechnologies |
| Alexa Fluor® 488 goat anti-mouse IgG (H+L) | IF (1:500) | | A11001, lifetechnologies |
| Anti-mouse IgG (H+L) | WB (1:10000) | | 042-06-18-06, KPL |
| anti-rabbit IgG (H+L) | WB (1:10000) | | 042-06-15-06, KPL |

Abbreviations: WB, Western blotting; IF, Immunofluorescence.
